# Autoimmune associations with chronic spontaneous urticaria: A retrospective cohort analysis

**DOI:** 10.1016/j.jdin.2025.05.002

**Published:** 2025-06-02

**Authors:** Dev Patel, Naeha Pathak, Omar Alani, Amit Singal, Shari R. Lipner

**Affiliations:** a Department of Dermatology, Icahn School of Medicine at Mount Sinai, New York, New York; b Department of Dermatology, Rutgers New Jersey Medical School, Newark, New Jersey; c Department of Dermatology, Weill Cornell Medicine, New York, New York

**Keywords:** urticaria, chronic urticaria, chronic spontaneous urticaria, autoimmune, comorbidities, TriNetX

*To the Editor:* Chronic spontaneous urticaria (CSU) is a mast cell-driven disease characterized by wheals and/or angioedema lasting >6-weeks. An estimated 10% to 28% of patients have autoimmune disease.^1^, ^2^, ^3^ We analyzed for risks of developing autoimmune comorbidities in CSU patients using a large multicenter database to help guide CSU workups.

On January 2, 24, TriNetX global research database was queried for patients ≥18-years with CSU diagnosis (ICD-10 L50.1). Patients with vs without CSU controls were propensity score-matched based on age, sex, race and ethnicity. Odds ratios of developing autoimmune diseases >1-day of CSU diagnosis were calculated. Follow-up for both cohorts were the same.

Overall, 105,187 CSU patients and 105,187 matched-controls were included. After matching, both CSU patients and controls were clinically similar (Table I). CSU patients vs controls had higher odds of developing vitiligo (odds ratio = 2.619, 95%CI 2.176-3.152), Sjögren's syndrome (2.369, 2.140-2.622), autoimmune thyroiditis (2.200, 2.057-2.353, pernicious anemia (2.154, 1.785-2.599), systemic lupus erythematous (SLE) (2.084, 1.879-2.310), dermatopolymyositis (2.120, 1.555-2.890), myasthenia gravis (2.001, 1.496-2.678), celiac disease (1.916, 1.70-2.16), Graves’ disease (1.879, 1.669-2.115), fibromyalgia (1.864, 1.770-1.963), ankylosing spondylitis (1.831, 1.496-2.241), psoriatic arthritis (1.376, 1.306-1.45), systemic sclerosis (1.348, 1.029-1.766), polymyalgia rheumatica (1.669, 1.399-1.991), inflammatory bowel disease (1.440, 1.360-1.524), psoriasis (1.321, 1.232-1.417), rheumatoid arthritis (1.313, 1.167-1.478), and type 1 diabetes mellitus (1.293, 1.191-1.404) (Table II).

**Table I:** Baseline characteristics of CSU patients and non-CSU patients before and after propensity score matching

Demographics	Before matching				After matching			
	CSU (*N* = 113,962)	No CSU (*N* = 12,239,816)	*P* value	SMD	CSU (*N* = 105,187)	No CSU (*N* = 105,187)	*P* value	SMD
Current age, mean ± SD	40.5 ± 22.3	53.2 ± 18.1	<.0001	0.626	42.1 ± 21.6	43.8 ± 19.4	<.0001	0.082
Male	32,009 (29.0%)	4,848,238 (40.4%)	<.0001	0.299	31,808 (30.2%)	24,611 (23.4%)	<.0001	0.155
Female	75,391 (68.3%)	6,467,001 (53.9%)	<.0001	0.104	70,441 (66.9%)	77,916 (74.1%)	<.0001	0.156
White	61,115 (55.4%)	7,253,712 (60.5%)	<.0001	0.104	59,686 (56.7%)	52,934 (50.3%)	<.0001	0.129
Black or African American	14,020 (12.7%)	1,647,579 (13.7%)	<.0001	0.031	13,857 (13.2%)	11,099 (10.6%)	<.0001	0.08
Asian	5911 (5.4%)	531,248 (4.4%)	<.0001	0.078	5646 (5.4%)	6588 (6.2%)	<.0001	0.04
Hispanic or Latino	11,147 (10.1%)	944,953 (7.9%)	<.0001	0.043	10,346 (9.8%)	14,903 (14.2%)	<.0001	0.13

ICD-10 Z00.00 was used as the index event for control patients.

*CSU*, Chronic spontaneous urticaria; *SD*, standard deviation; *SMD*, standard mean difference.

**Table II:** Odds of developing autoimmune comorbidities before and after propensity-score matching in patients with chronic urticaria: A TriNetX analysis

Autoimmune condition	OR (95% CI) before matching	OR (95% CI) after matching
Celiac disease	1.803 (1.681, 1.935)	1.916 (1.70,2.16)
Inflammatory bowel disease	1.038 (1.001, 1.076)	1.440 (1.360, 1.524)
Psoriasis	0.978 (0.934,1.024)	1.321 (1.232, 1.417)
Psoriatic arthritis	1.071 (0.972, 1.179)	1.376 (1.306, 1.45)
Sjögren's syndrome	1.867 (1.767, 1.974)	2.369 (2.140, 2.622)
Rheumatoid arthritis	1.004 (0.930, 1.084)	1.313 (1.167, 1.478)
Systemic lupus erythematous	2.057 (1.939, 2.182)	2.084 (1.879, 2.310)
Autoimmune thyroiditis	2.141 (2.061, 2.223)	2.200 (2.057, 2.353)
Graves’ disease	1.541 (1.437, 1.652)	1.879 (1.669, 2.115)
Type 1 diabetes mellitus	0.869 (0.823, 0.918)	1.293 (1.191, 1.404)
Vitiligo	1.864 (1.692, 2.053)	2.619 (2.176, 3.152)
Dermatopolymyositis	1.555 (1.308, 1.850)	2.120 (1.555, 2.890)
Myasthenia gravis	1.176 (0.994, 1.391)	2.001 (1.496, 2.678)
Systemic sclerosis	1.141 (0.957, 1.360)	1.348 (1.029, 1.766)
Pernicious anemia	1.252 (1.126, 1.391)	2.154 (1.785, 2.599)
Ankylosing spondylitis	1.289 (1.145, 1.452)	1.831 (1.496, 2.241)
Fibromyalgia	1.439 (1.395, 1.484)	1.864 (1.770, 1.963)
Polymyalgia rheumatica	0.898 (0.806, .00)	1.669 (1.399, 1.991)

ICD-10 K90.0 was used for celiac disease, K52.8 for inflammatory bowel disease, L40 for psoriasis, M05 for rheumatoid arthritis, M32 for systemic lupus erythematosus, E06.3 for autoimmune thyroiditis, E05.00 for Graves’ disease, E10 for type 1 diabetes mellitus, L80 for vitiligo, M33 for dermatopolymyositis, G70.0 for myasthenia gravis, M34.9 for systemic sclerosis, D51.0 for pernicious anemia, L40.50 for arthropathic psoriasis, M45 for ankylosing spondylitis, M79.7 for fibromyalgia, M35.3 for polymyalgia rheamatica, and M35.0 for Sjögren's Syndrome.

*CI*, Confidence interval; *OR*, odds ratio.

We found that CSU patients had increased odds of developing all assessed autoimmune comorbidities vs controls. A retrospective cohort study of 9332 CSU patients and 37,328 controls showed increased odds of SLE (1.58, 1.22-2.05), autoimmune thyroid disease (1.31, 1.20-1.43), Sjögren's syndrome (1.26, 1.01-1.57), and inflammatory bowel disease (1.22, 1.02-1.53), but decreased odds of psoriasis (0.54, 0.33-0.89).^1^ In a multicenter retrospective study analyzing 1999 CSU patients (no controls), 28% had ≥1 autoimmune disease, most commonly autoimmune thyroid disease (25%) and vitiligo (2%).^2^ In a population study of 12,778 CSU patients and 10,714 control patients, CSU patients had higher odds of rheumatoid arthritis (13.25, 7.39-23.76), type 1 diabetes mellitus (7.703, 4.78-12.65), Sjögren's syndrome (15.17, 5.54-14.54), celiac disease (26.96, 6.6-110.17), and SLE (14.59, 4.56-46.73).^3^ In contrast, a cohort study of 12,185 CSU patients and 104,007 controls reported no associations with type 1 diabetes mellitus (1.1, 0.9-1.4) and autoimmune thyroiditis (1.3, 0.7-2.6).^4^

The 2022 International Urticaria Guidelines support a limited workup for CSU, including annual thyroid function screening, and physical examination evaluating for signs of vitiligo and rheumatoid arthritis.^5^ Our study also highlights at least a review of systems for SLE, Sjögren's syndrome, dermatopolymyositis, ankylosing spondylitis, systemic sclerosis, and myasthenia gravis.

Limitations include inability to assess disease severity and multivariable linear regression, and potential miscoding. Matching was performed to reduce imbalance. TriNetX does not allow for multivariable adjustment or for determining time interval between CSU and autoimmune disease diagnosis. Observed odds ratios may not be clinically meaningful.

In conclusion, we found that CSU patients had increased odds of developing autoimmune comorbidities. Thus, we recommend a thorough history and review of systems, physical examination, and potential targeted laboratory workup, including antinuclear antibody, rheumatoid factor, and C-reactive protein to improve screening in these patients. Prospective studies examining the relationship between CSU and autoimmune diseases are an important area for future investigation.

## Conflicts of interest

None disclosed.
